# Association of *IL6* rs1800795, *TNF* rs1800629, *CCL2* rs1024611 and *VEGFA* rs699947 Polymorphisms with Bladder Cancer Risk, Tumor Aggressiveness, and HRV Parameters of Autonomic Nervous System Regulation

**DOI:** 10.3390/ijms27083361

**Published:** 2026-04-09

**Authors:** Vladimira Durmanova, Iveta Mikolaskova, Juraj Javor, Agata Ocenasova, Magda Suchankova, Boris Kollarik, Milan Zvarik, Maria Bucova, Luba Hunakova

**Affiliations:** 1 Institute of Immunology, Faculty of Medicine, Comenius University in Bratislava, 811 08 Bratislava, Slovakia; vladimira.durmanova@fmed.uniba.sk (V.D.); mikolaskova6@uniba.sk (I.M.); juraj.javor@fmed.uniba.sk (J.J.); agata.ocenasova@fmed.uniba.sk (A.O.); magda.suchankova@fmed.uniba.sk (M.S.); maria.bucova@fmed.uniba.sk (M.B.); 2 Department of Urology, Bory Hospital, 841 03 Bratislava, Slovakia; boris.kollarik@pentahospitals.com; 3 Department of Nuclear Physics and Biophysics, Faculty of Mathematics, Physics and Computer Science, Comenius University in Bratislava, 842 48 Bratislava, Slovakia; milan.zvarik@fmph.uniba.sk

**Keywords:** cytokines, polymorphism, inflammation, bladder cancer, tumor grade, heart rate variability

## Abstract

Chronic inflammation contributes to bladder cancer (BC) development and progression through dysregulated cytokine signaling and tumor–immune interactions. This case–control study investigated associations between *IL6* rs1800795, *TNF* rs1800629, *CCL2* rs1024611, and *VEGFA* rs699947 polymorphisms, circulating cytokine levels, clinicopathological characteristics, and autonomic nervous system balance assessed by heart rate variability (HRV) in 73 BC patients and 88 controls. Genotyping was performed using PCR–RFLP, serum cytokine levels were measured by ELISA, and associations were evaluated using logistic, linear regression, and survival analyses. No significant associations with BC risk were observed for *IL6*, *TNF*, or *VEGFA* variants. However, the *CCL2* rs1024611 GG genotype was associated with increased BC risk (recessive model: OR = 5.82, *p* = 0.026). Stratified analyses showed a lower frequency of the *IL6* rs1800795 C allele and *TNF* rs1800629 GA genotype in high-grade and muscle-invasive tumors, suggesting potential associations with reduced tumor aggressiveness. No polymorphism was associated with serum cytokine levels or disease-free survival. In BC patients, the *TNF* rs1800629 A allele was associated with higher parasympathetic-related HRV indices and lower sympathetic parameters, whereas no such associations were observed in controls. These findings indicate that genetic variation within inflammatory pathways may contribute to BC susceptibility and tumor phenotype and may also modulate neuroimmune interactions.

## 1. Introduction

Bladder cancer (BC) remains one of the most prevalent malignancies worldwide, accounting for approximately 3.1% of all newly diagnosed cancer cases in 2022 according to GLOBOCAN data [1]. More than 90% of BC cases are histologically classified as urothelial carcinomas [2]. Tumors that do not invade the muscle layer of the bladder are classified as Ta and T1 and are collectively referred to as non-muscle-invasive bladder cancer (NMIBC). In contrast, tumors classified as T2, T3, and T4 invade the detrusor muscle or beyond and are categorized as muscle-invasive bladder cancer (MIBC) [3]. Despite advances in the diagnosis and treatment, 30–80% of NMIBC patients experience recurrence, and 10–20% of cases progress to MIBC, where the 5-year survival rate drops to approximately 60% [4].

Chronic inflammation and the resulting inflammatory microenvironment represent major risk factors for cancer development [5,6]. In the bladder urothelium, persistent inflammation may result from recurrent urinary tract infections, exposure to carcinogens such as tobacco smoke and aromatic amines, or intravesical therapies including Bacillus Calmette–Guérin (BCG) instillation [7]. Sustained inflammation in the bladder mucosa triggers a cascade of immunological events, including the recruitment of innate immune cells, enhanced cytokine secretion, and epithelial stress responses, collectively disturbing tissue homeostasis and promoting oncogenic reprogramming [6]. Proinflammatory cytokines and chemokines, including interleukin (IL)-1, IL-6, tumor necrosis factor (TNF), and monocyte chemoattractant protein (MCP)-1, have been implicated in BC development and progression [8,9,10,11,12].

Interleukin-6 (IL-6) is a multifunctional cytokine exhibiting both pro- inflammatory and anti-inflammatory activities [13]. Through activation of STAT3 and PI3K/Akt signaling pathways, IL-6 promotes tumor cell proliferation, survival, and angiogenesis [14]. Overexpression of IL-6 has been associated with BC progression, recurrence, metastasis, and reduced overall survival in BC patients [11,12,15,16,17]. The rs1800795 (−174 G>C) polymorphism located in the promoter region of the *IL6* gene influences transcriptional activity and IL-6 expression levels [18]. However, studies evaluating its association with BC susceptibility have yielded inconsistent results across populations [19,20,21,22,23].

Tumor necrosis factor (TNF) is a pro-inflammatory cytokine that exhibits context-dependent dual roles in tumor biology. In early tumorigenesis, high local TNF levels may induce apoptosis of malignant cells, enhance dendritic cell (DC) maturation, upregulate antigen-presenting cell (APC) machinery, suppress T regulatory cell (Treg) function, stimulate neutrophils and M1 macrophages to mount anti-tumor immune responses, and disrupt tumor vascularization [24,25,26]. Conversely, in a chronically inflamed tumor microenvironment (TME), TNF preferentially activates NF-κB and STAT3 signaling pathways, thereby promoting angiogenesis, epithelial–mesenchymal transition (EMT), invasion, and recruitment of immunosuppressive myeloid-derived suppressor cells (MDSCs) [26]. Increased TNF expression has been documented in both tumor and stromal compartments of multiple malignancies [27,28,29,30,31]. In human BC cells, TNF induces the secretion of matrix metalloproteinase-9 (MMP-9), thereby facilitating tumor invasion and metastasis [32]. Moreover, TNF overexpression has been associated with angiogenesis and disease progression in bladder carcinomas [8]. The promoter variant *TNF* rs1800629 (−308 G>A) enhances TNF transcription and protein production [33,34] and has been associated with increased BC risk and invasive disease in some studies [35,36,37].

Monocyte chemoattractant protein-1 (MCP-1), also known as the chemokine (C-C motif) ligand 2 (CCL2), is a key chemokine regulating immune cell recruitment within the TME. Through activation of the CCR2 receptor and downstream PI3K/Akt and MAPK pathways, CCL2 promotes tumor cell proliferation, migration, and EMT, while simultaneously recruiting tumor-associated macrophages (TAMs) and MDSCs [38]. Increased CCL2 expression has been observed in BC tissues and human BC cell lines [39] and correlates with tumor invasion, disease progression, and lymphatic metastasis [9,40]. The rs1024611 (−2518 A>G) polymorphism located in the promoter region of the *CCL2* gene has been associated with increased CCL2 expression [41] and poor clinical prognosis in several malignancies [38].

Vascular endothelial growth factor A (VEGF-A) is a key regulator of vascular endothelial cell proliferation and angiogenesis. It promotes tumor vascularization, cancer cell proliferation, invasion, and migration [42]. Beyond its vascular effects, VEGF-A modulates antitumor immunity within the TME by impairing DC maturation, promoting Treg expansion and accumulation, and enhancing expression of inhibitory immune checkpoints such as PD-1, CTLA-4, TIM-3, and LAG-3 on CD8^+^ T cells [43,44,45]. Increased VEGF-A expression has been associated with advanced disease stage and poor prognosis in patients with BC [46,47,48,49]. The promoter *VEGFA* rs699947 (−2578 C>A) polymorphism influences transcriptional activity, with the C allele linked to increased VEGF-A expression [50,51]. Case-control studies and meta-analyses investigating the association between *VEGFA* rs699947 and BC risk have yielded inconsistent results across different populations [52,53,54,55].

Growing evidence suggests bidirectional interactions between inflammatory pathways and autonomic nervous system (ANS) regulation. Time- and frequency-domain analyses of heart rate variability (HRV) represent non-invasive methods for assessing the balance between the parasympathetic (vagal) and sympathetic branches of ANS [56,57,58]. Reduced vagal activity and sympathetic predominance may contribute to systemic inflammation [59,60,61,62]. One mechanism underlying this relationship is the vagally mediated reflex, known as the cholinergic anti-inflammatory pathway, through which diminished parasympathetic activation may enhance inflammatory responses [61,63,64]. Conversely, inflammatory and immunological signals may modulate parasympathetic vagal activity. In healthy individuals, lower vagal tone has been independently associated with increased circulating levels of IL-6 and TNF [65,66,67,68]. Moreover, negative correlations between parasympathetic HRV indices and inflammatory markers, including TNF, IL-6, IL-1, and C-reactive protein (CRP), have been reported in multiple pathological conditions [60,69].

Although inflammatory cytokine gene polymorphisms have been studied in relation to BC susceptibility, their potential influence on autonomic regulation and neuroimmune interactions in BC patients remains unexplored. To our knowledge, no previous study has simultaneously evaluated inflammatory cytokine gene variants, circulating cytokine levels, clinical characteristics, and HRV-derived ANS parameters in BC. Therefore, the primary objective of this study was to investigate the association between *IL6* rs1800795, *TNF* rs1800629, *CCL2* rs1024611, and *VEGFA* rs699947 polymorphisms, their corresponding serum cytokine levels, and clinicopathological parameters in BC patients. The secondary objective was to assess whether genetic variation within inflammatory pathways is associated with ANS balance as indexed by HRV.

## 2. Results

### 2.1. Characteristics of the Study Groups

The baseline characteristics of the study population are presented in Table 1. The study included 73 BC patients and 88 control subjects. A statistically significant difference in sex distribution was observed between the BC group and controls (*p* = 0.02), with a higher proportion of men among BC patients compared with controls (68.49% vs. 50.00%). A significant difference between the groups was also detected with respect to age at examination (*p* = 0.0001). The majority of BC patients had a positive smoking history (64.38%), whereas only a minority of control subjects were smokers (34.09%; *p* = 0.0001). Statistically significant differences in BMI values were found, with BC patients having higher BMI compared to controls (*p* = 0.04). Among the 73 BC patients, 31 were diagnosed with low-grade tumors and 42 with high-grade tumors. Based on the depth of tumor invasion, patients were classified as having non-muscle-invasive bladder cancer (NMIBC), including pTa/CIS (*n* = 29) and pT1 (n = 28) tumors, or muscle-invasive bladder cancer (MIBC), comprising pT2 (n = 12) and pT3 (n = 4) tumors. Mean disease-free survival (DFS) time was 18.11 ± 11.21 months.

### 2.2. Association of Cytokine Gene Polymorphisms with BC Risk

Allele and genotype frequencies of cytokine gene polymorphisms in BC group and controls are shown in Table 2. The genotype distribution of the majority of cytokine gene polymorphisms fit the HWE in our BC patients (*IL6* rs1800795: χ^2^ = 0.21, *p* = 0.64, *VEGFA* rs699947: χ^2^ = 0.03, *p* = 0.86) as well as in controls (*IL6* rs1800795: χ^2^ = 0.41, *p* = 0.52, *TNF* rs1800629: χ^2^ = 0.77, *p* = 0.38, *CCL2* rs1024611: χ^2^ = 0.77, *p* = 0.38). However, a significant departure from HWE in BC patients was observed for *TNF* rs1800629 (χ^2^ = 5.60, *p* = 0.02) and *CCL2* rs1024611 (χ^2^ = 4.20, *p* = 0.04), whereas *VEGFA* rs699947 deviated from HWE in controls (χ^2^ = 7.36, *p* = 0.01).

Allelic comparison of the *IL6* rs1800795, *TNF* rs1800629, and *VEGFA* rs699947 polymorphisms revealed no statistically significant differences between the BC and control groups. Consistent with these findings, logistic regression analysis adjusted for potential confounders demonstrated no association between any of the three SNPs and the risk of BC under any of the inheritance models examined (*p* > 0.05, Table 2). Although no differences in allele distribution were observed for the *CCL2* rs1024611 polymorphism between BC patients and controls, genotype-based analysis revealed a significantly increased risk of BC for the mutant GG genotype under codominant (GG vs. AA: OR = 5.40, *p* = 0.029) and recessive genetic models (GG vs. AA + AG: OR = 5.82, *p* = 0.026) (Table 2).

### 2.3. Association of Cytokine Gene Polymorphisms with Serum Cytokine Level and Clinical Variables in BC Patients

Linear and logistic regression analyses were performed to assess the association between cytokine gene polymorphism and serum cytokine levels, as well as clinicopathological variables, including tumor grade and stage, in BC patients. No statistically significant associations were observed between the *IL6* rs1800795, *TNF* rs1800629, *CCL2* rs1024611, or *VEGFA* rs699947 genotypes and serum cytokine levels in BC patients (*p* > 0.05, Supplementary Table S1).

Stratification of the BC group according to tumor grade revealed a significantly lower frequency of *IL6* rs1800795 C allele and CC mutant genotype in patients with high-grade tumors compared with those with low-grade tumors (C vs. G allele contrast: OR = 0.51, *p* = 0.049; CC vs. GG + GC recessive model: OR = 0.30, *p* = 0.049, Table 3). In the analysis of *TNF* rs1800629, a lower frequency of the GA genotype was observed in patients with high-grade tumors compared with those with low-grade tumors. This difference was reflected in significantly reduced odds ratios under the codominant (GA vs. GG: OR = 0.23, *p* = 0.025), dominant (GA + AA vs. GG: OR = 0.30, *p* = 0.041), and overdominant (GA vs. GG + AA; OR = 0.23, *p* = 0.025) genetic models (Table 3). Analysis of *CCL2* rs1024611 and *VEGFA* rs699947 polymorphisms revealed no statistically significant differences in allele and genotype distribution between the two groups (*p* > 0.05, Table 3).

Stratification of BC group according to tumor stage revealed a significantly lower frequency of the *IL6* rs1800795 C allele carriers among the patients with pT2 or pT3 stage tumors when compared to pTa or pT1 stage patients (GC + CC vs. GG dominant model: OR = 0.28, *p* = 0.034; log-additive model: OR = 0.42, *p* = 0.047, Table 4). The analysis of *TNF* rs1800629 SNP revealed a significantly decreased frequency of the GA genotype in pT2 + pT3 group when compared to pTa + pT1 group (GA vs. GG codominant model: OR = 0.17, *p* = 0.045; GA vs. GG + AA overdominant model: OR = 0.16, *p* = 0.048). Furthermore, there was a significantly lower frequency of *CCL2* rs1024611 G allele in patients with pT2 or pT3 stage tumors when compared to pTa + pT1 group (G vs. A allele contrast: OR = 0.20, *p* = 0.007; AG + GG vs. AA dominant model: OR = 0.25, *p* = 0.032; log-additive model: OR = 0.27, *p* = 0.013, Table 4). On the other hand, no statistically significant link between *VEGF* rs699947 and tumor stage was found in BC patients (*p* > 0.05, Table 4).

### 2.4. Association of Cytokine Gene Polymorphisms with Disease-Free Survival Rates

**Figure 1:**
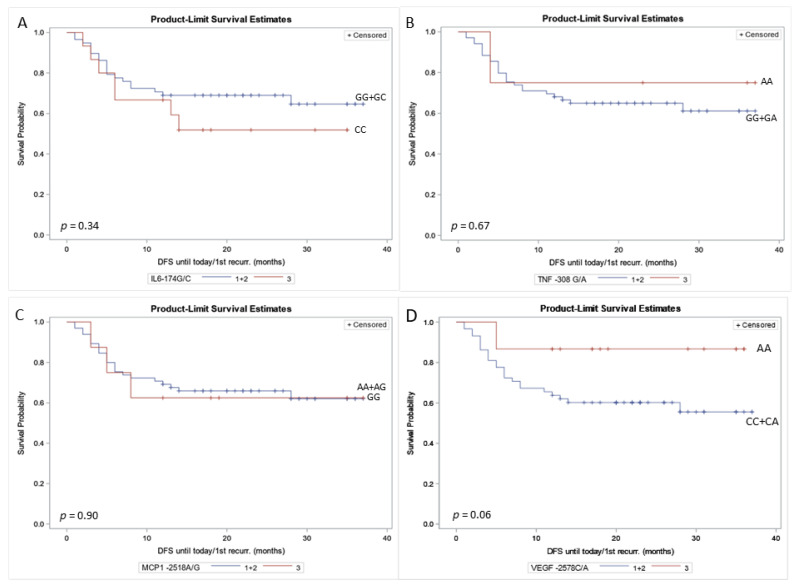
Association of cytokine gene polymorphism with DFS in BC patients. Kaplan–Meier estimates of disease-free survival according to *IL6* rs1800795 (−174 G/C) (**A**), *TNF* rs1800629 (−308 G/A) (**B**), *MCP1* rs1024611 (−2518 A/G) (**C**), and *VEGFA* rs699947 (−2578 C/A) genotypes (**D**). X-axis: DFS (months); Y-axis: survival probability, Abbreviations: DFS: disease-free survival; recurr: recurrence; + Censored, recurrence-censored data.

Differences in DFS according to cytokine genotypes were evaluated using Kaplan–Meier analysis with the log-rank test for group comparisons. No significant associations were observed between cytokine gene polymorphisms and DFS. Specifically, mean DFS was 15.53 ± 11.15 months for *IL6* rs1800795 CC carriers compared with 18.78 ± 11.23 months for GC + GG carriers (log-rank *p* = 0.34). For *TNF* rs1800629, mean DFS was 25.00 ± 15.38 months for AA carriers versus 17.71 ± 10.94 months for GA + GG carriers (log-rank *p* = 0.67). In the case of *CCL2* rs1024611, mean DFS was 17.13 ± 12.96 months for GG carriers and 18.23 ± 11.09 months for AG + AA carriers (log-rank *p* = 0.90). For *VEGFA* rs699947, mean DFS was 21.33 ± 11.30 months for AA carriers compared with 17.28 ± 11.14 months for CA + CC carriers (log-rank *p* = 0.06) (Figure 1).

### 2.5. Association of Cytokine Gene Polymorphisms with HRV Parameters

In the BC group, the mutant A allele of *TNF* rs1800629 was associated with higher predominantly parasympathetic HRV parameters, including SDNN (log-additive model: *p* = 0.0066), RMSSD (recessive model: *p* = 0.011), DCmod (recessive model: *p* = 0.013), the PNS index (log-additive model: *p* = 0.016; Table 5). An association was also observed with a parameter reflecting global heart rate variability influenced by both autonomic branches, namely total power (recessive model: *p* = 0.0008). Furthermore, the A allele was associated with lower sympathetic or stress-related parameters, including ACmod (log-additive model: *p* = 0.021), mean HR (dominant model: *p* = 0.040), the SNS index (dominant model: *p* = 0.021), and the stress index (dominant model: *p* = 0.035; Table 5). In contrast, no associations between *TNF* rs1800629 and any of the above-mentioned HRV parameters were observed in the control group (*p* > 0.05; Supplementary Table S2).

Moreover, no significant associations were found between *IL6* rs1800795, *CCL2* rs1024611, or *VEGFA* rs699947 polymorphisms and HRV parameters in BC patients (Supplementary Tables S3–S5).

## 3. Discussion

Emerging evidence suggests that chronic inflammation contributes to bladder carcinogenesis through sustained cytokine signaling, immune dysregulation, and tumor–microenvironment interactions [5,6,70]. Our previous study by Mikolaskova et al. [71] directly compared selected inflammatory markers between bladder cancer (BC) patients and healthy controls and demonstrated a distinct inflammatory profile in the BC group. BC patients showed increased levels of TREM-1/TREM-2, sTREM-1, the sTREM-1/sTREM-2 ratio, BDNF, MCP-1, and NLR, and reduced levels of IFN-γ, IL-10, LMR, and PMR compared with controls [71].

In this study, we investigated functional promoter polymorphisms in key inflammatory genes (*IL6*, *TNF*, *CCL2*, *VEGFA*) and evaluated their associations with BC susceptibility, clinicopathological characteristics, circulating cytokine levels, and autonomic nervous system regulation assessed by heart rate variability.

We did not observe significant associations between *IL6* rs1800795, *TNF* rs1800629, or *VEGFA* rs699947 polymorphisms and overall BC risk. In contrast, the *CCL2* rs1024611 GG genotype was associated with increased BC susceptibility in our cohort. Although none of the investigated polymorphisms were related to circulating cytokine levels or disease-free survival, stratified analyses indicated that *IL6* rs1800795, *TNF* rs1800629, and *CCL2* rs1024611 variants were associated with clinicopathological features suggestive of reduced tumor aggressiveness. Additionally, *TNF* rs1800629 A allele was linked to higher parasympathetic-related HRV indices in BC patients, supporting a potential interaction between inflammatory genetic variation and ANS regulation.

Interleukin-6 (IL-6) is a multifunctional cytokine involved in immune regulation and hematopoiesis, exerting context-dependent pro- or anti-inflammatory effects [13]. By promoting cell proliferation, survival, and resistance to apoptosis, IL-6 has been implicated in tumorigenesis [14], and elevated IL-6 expression has been associated with poorer prognosis and reduced survival in BC patients [11,12,15,16,17]. The *IL6* rs1800795 C allele at position −174 (G>C) has been linked to lower transcriptional activity and IL-6 production [18]; however, its impact on BC susceptibility remains controversial [19,20,21,22]. A recent meta-analysis reported significant association of the C allele with increased BC risk predominantly in Asian populations, but not in Caucasians [23]. Consistent with these findings, we observed no significant association between *IL6* rs1800795 and overall BC risk in our cohort. Moreover, while we observed no influence of the polymorphism on circulating IL-6 levels or DFS, the C allele was significantly less frequent in high-grade and muscle invasive tumors, suggesting a potential protective effect of low-producing *IL6* rs1800795 C allele against tumor aggressiveness. The absence of genotype-dependent differences in serum IL-6 concentrations indicates that local TME regulation, rather than systemic cytokine levels, may underlie these associations.

Tumor necrosis factor is a central pro-inflammatory cytokine with both pro- and anti-tumorigenic properties [8,24,25,26,32]. Elevated circulating TNF levels have been associated with BC development in some studies [8,72]. The rs1800629 (−308 G>A) variant in the promoter region of the *TNF* gene influences gene expression, with the A allele reported to enhance TNF production [33,34,73]. Previous case–control studies examining its association with BC susceptibility have yielded conflicting result [21,35,36,72], and meta-analyses have generally not supported a significant role of this variant in overall BC risk [37,74,75]. Consistent with these data, we observed no association between *TNF* rs1800629 and BC risk in our cohort. However, stratified analyses revealed a significantly higher frequency of the *TNF* rs1800629 GA genotype in low-grade and non-invasive tumors. Interestingly, while some reports have linked rs1800629 to increased tumor invasiveness and higher grade [37,72], others have suggested a protective effect in less aggressive disease [75]. Given the transcription-enhancing effect of the A allele, our findings may reflect a context-dependent role of TNF signaling in early tumor immunosurveillance. Importantly, rs1800629 was not associated with circulating TNF levels or DFS, suggesting limited systemic or prognostic impact within this cohort. Overall, our data indicate that the *TNF* rs1800629 variant may modulate tumor aggressiveness rather than overall BC susceptibility, with the high-producing GA genotype exerting a protective effect [73].

Monocyte chemoattractant protein-1 (MCP-1), also known as CCL2, is a chemokine that regulates immune cell recruitment within the TME and promotes cancer cell proliferation, migration, and EMT [38]. The promoter polymorphism *CCL2* rs1024611 (−2518 A>G) influences transcriptional activity, with the G allele associated with enhanced CCL2 expression and increased monocyte recruitment [41], potentially amplifying tumor-promoting inflammation and disease progression in a variety of cancers [38]. In our study, the GG genotype was significantly associated with higher BC risk, supporting a role for CCL2-mediated inflammatory signaling in disease susceptibility. Although previous investigations have reported inconsistent findings regarding *CCL2* rs1024611 and BC susceptibility [76,77,78,79,80], meta-analytic evidence supports an association between the GG genotype and increased BC risk in Caucasian populations [81], consistent with our findings. Notably, stratification of our BC patients according to tumor stage suggested that the effect of the G allele may be more pronounced in non-invasive tumors, implying a potential role in early tumor development. Given the heterogeneity of prior reports concerning tumor grade and stage [76,79], larger studies incorporating functional analyses are warranted to clarify the complex effects of *CCL2* rs1024611 in BC development.

Vascular endothelial growth factor A (VEGF-A) plays a central role in angiogenesis and tumor vascularization and additionally modulates antitumor immunity, cancer cell proliferation, invasion, and migration [42,43,44,45]. Increased tissue expression and circulating levels of VEGF-A have been reported in patients with BC [82,83], supporting its involvement in BC development and progression [46,47,48]. Genetic variants within regulatory regions of the *VEGFA* gene have therefore been investigated as potential modifiers of BC risk, with several large-scale case–control studies and meta-analyses suggesting associations between selected variants and disease susceptibility [54,84,85]. The promoter variant rs699947 (−2578 C>A) has been reported to influence transcriptional activity, with some studies suggesting higher VEGF-A expression associated with the C allele [50,51]. In the present study, we did not observe a significant association between *VEGFA* rs699947 and overall BC risk. This finding is consistent with meta-analyses indicating that the relationship between rs699947 and BC susceptibility may be population-specific and more evident in Asian cohorts [53,55]. Furthermore, we found no association between *VEGFA* rs699947 and tumor grade or stage, suggesting limited impact on disease aggressiveness in our population. In contrast, some previous analyses have reported a protective association of the A allele with muscle-invasive BC [54], highlighting potential ethnic or context-dependent effects. Collectively, our results do not support a major role of *VEGFA* rs699947 in BC susceptibility or progression within this cohort.

To the best of our knowledge, *IL6* rs1800795, *TNF* rs1800629, *CCL2* rs1024611, and *VEGFA* rs699947 polymorphisms have not previously been evaluated in relation to disease-free survival in BC. In our cohort, none of the investigated variants showed a significant association with DFS. Nevertheless, survival-modifying effects of these polymorphisms have been reported in other malignancies. The *IL6* rs1800795 GG genotype has been associated with reduced overall survival in advanced gastric cancer [86] and pediatric neuroblastoma [87]. Furthermore, the C allele has been linked to 5-year overall survival in breast cancer [88]. Similarly, the *TNF* rs1800629 AA genotype has been associated with shorter event-free survival in pediatric neuroblastoma patients [89]. For *CCL2* rs1024611, non-AA genotypes have been correlated with decreased survival in colorectal cancer [90]. Regarding *VEGFA* rs699947, the A allele have been associated with shorter progression-free survival in sunitinib-treated metastatic renal cell carcinoma patients [91] and shorter DFS among breast-cancer patients treated with neoadjuvant chemotherapy followed by mastectomy [92]. Finally, the *VEGFA* rs699947 AA genotype was associated with shorter progression-free survival in patients with metastatic colorectal cancer [93]. These findings suggest that the prognostic relevance of inflammatory gene polymorphisms may be tumor-type specific and influenced by treatment context.

A novel aspect of our study is the evaluation of cytokine gene variants in relation to ANS regulation. High HRV is generally considered a marker of balanced ANS function, greater stress resilience, and better overall health, whereas reduced HRV has been associated with stress, anxiety, and various pathological conditions [94]. In our previous analysis of BC patients, we observed a pattern of sympathetic predominance, reflected by higher SNS index, stress index, and ACmod values, accompanied by reduced parasympathetic modulation, as indicated by lower PNS index, SDNN, RMSSD, and DCmod compared with controls [71]. Experimental and clinical evidence suggests that parasympathetic activity may exert anti-inflammatory effects through the vagally mediated cholinergic anti-inflammatory pathway [63], whereas sympathetic activation has been linked to enhanced inflammatory responses [95]. Supporting this concept, a 2019 meta-analysis demonstrated significant negative correlations between vagally mediated HRV indices and inflammatory markers, including TNF, IL-6, IL-1, and CRP [60]. Another study reported significant negative correlations between high frequency (HF) HRV indices and neutrophil-to-lymphocyte ratio (NLR), systemic immune-inflammation index (SII), CRP, and IL-6, demonstrating an association between reduced vagally mediated HRV and elevated systemic inflammation in patients with COVID-19 [96]. A positive correlation between sympathetic HRV indices and soluble triggering receptor expressed on myeloid cells-1 (sTREM-1), the sTREM-1/sTREM-2 ratio, fractalkine, and inflammatory markers including the SII, NLR, and platelet-to-lymphocyte ratio (PLR), together with a negative correlation with parasympathetic HRV indices, has been reported in BC patients [71]. Mechanistically, these findings converge toward a model in which autonomic imbalance reflects and potentially modulates the inflammatory milieu present in cancer. Parasympathetic activation can suppress pro-inflammatory cytokine release via α7-nicotinic receptor–mediated signaling, whereas sympathetic activity may amplify inflammatory cascades through catecholamine-driven pathways [63]. This bidirectional neuroimmune cross-talk provides a biological rationale for interpreting HRV alterations as markers of tumor-related inflammatory activity rather than merely indicators of generalized stress physiology.

In our BC cohort, the *TNF* rs1800629 A allele was associated with higher parasympathetic-related HRV indices (RMSSD, DCmod, PNS index) and lower sympathetic-related parameters (mean HR, SNS index, stress index), whereas no such associations were observed in controls. These findings suggest that TNF genetic variation may modulate neuroimmune interactions specifically within the tumor context. Given that the rs1800629 A allele has been linked to enhanced TNF transcriptional activity, its association with increased parasympathetic-related HRV parameters and lower sympathetic tone may appear paradoxical at first glance. One possible explanation is that enhanced vagal activity in A allele carriers reflects a compensatory or regulatory response within a pro-inflammatory environment, potentially engaging cholinergic anti-inflammatory pathways. The absence of genotype-dependent changes in circulating TNF levels suggests that these effects may occur at a local or signaling-network level rather than through systemic cytokine concentration shifts. This interpretation aligns with our observation that *TNF* rs1800629 was more frequent in less aggressive tumors, supporting a context-dependent role of TNF signaling in BC progression.

These genotype-specific autonomic patterns may have direct translational relevance. First, HRV could serve as a non-invasive indicator of individual inflammatory responsiveness modulated by cytokine gene variants. Second, differences in autonomic balance may contribute to variability in disease progression, treatment tolerance, or stress resilience among BC patients. Third, our findings raise the possibility that patients with certain inflammatory genotypes—such as *TNF* rs1800629 A carriers—might differentially benefit from interventions targeting autonomic regulation, including vagal nerve stimulation, mind–body therapies, and physical activity programs known to enhance parasympathetic tone. Finally, integrating HRV, inflammatory genotyping, and tumor characteristics may improve patient stratification and support the development of personalized therapeutic strategies focused on the neuroimmune axis.

Several limitations of the present study should be acknowledged. First, the relatively modest sample size may have limited statistical power, particularly in stratified analyses according to tumor grade and stage, as well as in survival analyses. Second, deviations from HWE were observed for selected polymorphisms in certain groups. Although such deviations can occur due to chance in smaller cohorts, population stratification, or disease association, these findings should be interpreted with caution. Replication in larger cohorts is necessary to confirm the robustness of associations observed in our study. Third, we evaluated circulating cytokine concentrations as systemic biomarkers; however, serum levels may not accurately reflect local cytokine activity within the TME. Functional assays evaluating gene expression in tumor tissue, immune cell subsets, or in vitro transcriptional activity would provide better insight into the biological consequences of the investigated promoter polymorphisms. In addition, although we adjusted our analyses for major confounders (age, sex, BMI, and smoking), residual confounding by environmental exposures, treatment heterogeneity, and comorbid conditions cannot be excluded.

Overall, our findings provide preliminary indications that inflammatory genetic variation may play a role in modulating BC susceptibility, and tumor characteristics, while also influencing neuroimmune interactions relevant to autonomic regulation.

## 4. Materials and Methods

### 4.1. Study Groups

This study enrolled 73 BC patients (50 males and 23 females) who underwent partial or complete resection of bladder tumors. The average age of BC patients was 67.00 ± 8.99 years. Patients were randomly recruited from the Department of Urology, St. Cyril and Methodius Hospital Bratislava, Slovakia. The diagnosis was approved by two pathologists according to the most recent WHO classification criteria [97]. The control group comprised 88 unrelated individuals (44 males and 44 females) with a mean age of 60.08 ± 13.07 years. Control subjects were randomly recruited from a broader population cohort. Those individuals who reported personal or family history of cancer and/or ongoing or recent acute inflammatory condition were excluded from the study. All participants provided written informed consent to be enrolled in this study and for personal data management. The study was conducted in accordance with the International Ethical Guidelines and the Declaration of Helsinki and was approved by the Ethical Committees of both the Faculty of Medicine, Comenius University, and University Hospital in Bratislava, Slovakia (approval code: 125/2021), and St. Cyril and Methodius Hospital, Bratislava (approval code: EK/1/2/2022).

### 4.2. Genotyping

Both patient and control DNAs were isolated from EDTA-treated whole blood by a modified salting out procedure [98].

The *IL6* rs1800795 (−174 G>C) polymorphism was genotyped by polymerase chain reaction (PCR) followed by restriction fragment length polymorphism (RFLP) analysis according to the protocol by Wu et al. (2013) [36]. Amplification using specific primers generated a 198 bp PCR product, which was subsequently digested with the restriction enzyme SfaNI (New England Biolabs, Ipswich, Massachusetts, USA). The C allele yielded an intact 198 bp fragment, whereas the G allele produced two fragments of 140 bp and 58 bp.

Genotyping of the *TNF* rs1800629 (−308 G>A) variant was performed by PCR-RFLP as previously described by Wu et al. (2013) [36]. A 107 bp product was generated by PCR and subjected to enzymatic digestion with NcoI (New England Biolabs, Ipswich, Massachusetts, USA). The presence of the A allele was indicated by an undigested fragment, while digestion of the G allele produced fragments of 87 bp and 20 bp.

The *CCL2* rs1024611 (−2518 A>G) polymorphism was analyzed following the PCR-RFLP protocol reported by Bagci et al. (2015) [99]. After amplification of a 234 bp target sequence, restriction digestion was carried out using PvuII (New England Biolabs, Ipswich, Massachusetts, USA). Samples carrying the A allele displayed an intact PCR product, whereas the G allele was identified by the appearance of two fragments measuring 159 bp and 75 bp.

For the *VEGFA* rs699947 (−2578 C>A) polymorphism, genotyping was conducted by PCR-RFLP in accordance with Sajjadi et al. (2020) [100]. The amplified 325 bp PCR product was digested with BglII (New England Biolabs, Ipswich, Massachusetts, USA). While the C allele was not cleaved by the enzyme, digestion of the A allele yielded two fragments of 202 bp and 123 bp.

### 4.3. Cytokine Level Analysis

Serum was obtained by centrifugation of 5 mL of clotted blood samples of BC patients on the day of chirurgical treatment. Serum was obtained from healthy control subjects as well. The serum levels of cytokines IL-6, TNF, CCL2, and VEGF-A were determined by sandwich ELISA assay kits according to the manufacturer’s instructions (Fine Biotech Co., Ltd., Wuhan, China). Briefly, diluted samples and standards were added to the microplate wells and incubated for 90 min at 37 °C. After washing, the biotin-labeled antibody was added and incubated for 60 min at 37 °C. Following additional washing steps, the HRP-streptavidin conjugate was added and incubated for 30 min at 37 °C. Finally, TMB substrate was added and allowed to react for 15 min at 37 °C. The reaction was stopped by adding stop solution to each well, and the absorbance at 450 nm was measured using a microplate reader.

### 4.4. Measurement of HRV Parameters

HRV parameters known to reflect sympathetic and parasympathetic activity were selected and analyzed as previously described [101]. All recordings were obtained under standardized resting conditions, with participants seated in a quiet environment and a minimum of 6 min of continuous ECG measurement with a 1000 Hz sampling rate. Sympathetic and stress-related indices included time-domain measures of mean heart rate (HR, beats/min), modified acceleration capacity of the heart rate (ACmod, ms), the sympathetic nervous system (SNS) index, and the stress index. Parasympathetic HRV parameters included the time-domain parameters of the standard deviation of normal-to-normal interbeat intervals (SDNN, ms), root mean square of successive differences between normal heartbeats (RMSSD, ms), modified deceleration capacity of the heart rate (DCmod, ms), and the parasympathetic nervous system (PNS) index. Artefact correction was performed using validated automated algorithms with manual inspection to ensure data quality. The other parameter was the total power as a marker of the overall autonomic activity. All indices were computed using the HRV analysis Kubios Premium software, version 3.5.0 (Kubios Oy, Kuopio, Finland) according to established guidelines.

### 4.5. Statistical Analysis

Comparisons of categorical variables between study groups were performed using the χ^2^ test, whereas differences in continuous variables were assessed with the Mann–Whitney U test. SNP genotype distributions were evaluated for deviation from Hardy–Weinberg equilibrium (HWE) using the χ^2^ goodness-of-fit test with one degree of freedom. Differences in allele frequencies between BC patients and controls, between low- and high-grade tumor groups, and between pTa + pT1 and pT2 + pT3 tumor stages were analyzed using the Pearson χ^2^ test in InStat statistical software (GraphPad Software version 3.0., Inc., San Diego, CA, USA). Logistic regression analysis adjusted for potential confounders (age, sex, BMI and smoking status) was employed to assess associations between individual genotypes, and BC risk, tumor grade, and stage. Odds ratios (ORs), 95% confidence intervals (95% CIs), and corresponding *p*-values were calculated under codominant, dominant, recessive, over-dominant and log-additive inheritance models using SNPstats web software available at https://www.snpstats.net/start.htm (accessed on 14 February 2025) [102]. Associations between cytokine SNPs and serum cytokine levels as well as HRV parameters were evaluated by linear regression analysis. Disease-free survival according to genotype was analyzed using the Kaplan–Meier method with the log-rank test. DFS was defined as the time from the date of primary treatment to the first occurrence of tumor recurrence. A *p*-value of <0.05 was considered statistically significant.

## 5. Conclusions

This case–control study demonstrates that the *CCL2* rs1024611 GG genotype is associated with increased bladder cancer risk, while *IL6* rs1800795 and *TNF* rs1800629 variants may be linked to reduced tumor aggressiveness rather than disease susceptibility. No associations were observed with circulating cytokine levels or disease-free survival. In patients with bladder cancer, the *TNF* rs1800629 A allele was associated with increased parasympathetic related heart rate variability (HRV) indices and reduced sympathetic HRV parameters. Notably, these autonomic associations were absent in the control group. Collectively, these findings indicate that genetic variation within inflammatory pathways may contribute to bladder cancer susceptibility and tumor phenotype, while also modulating neuroimmune interactions relevant to autonomic regulation. Our study underscores the multifaceted role of cytokine gene polymorphisms in bladder cancer and supports continued investigation into neuroimmune mechanisms involved in tumor biology.

## Figures and Tables

**Table 1:** Characteristics of the studied groups.

Parameter	Bladder Cancer (n = 73)	Controls (n = 88)	*p*-Value
Gender ratio males/females	50 (68.49%)/23 (31.51%)	44 (50.00%)/44 (50.00%)	**0.02**
Age at examination (mean ± SD)	67.00 ± 8.99	60.08 ± 13.07	**0.0001**
Smoking			
No	26 (35.62%)	58 (65.91%)	
Yes	47 (64.38%)	30 (34.09%)	**0.0001**
BMI	27.65 ± 4.65	26.30 ± 3.69	**0.04**
Grade			
Low	31 (42.47%)	-	
High	42 (57.53%)	-	
Tumor staging			
pTa/CIS	29 (39.73%)	-	
pT1	28 (38.35%)	-	
pT2	12 (16.44%)	-	
pT3	4 (5.48%)	-	
Disease free survival (mean ± SD, months)	18.11 ± 11.21	-	
Low grade	19.58 ± 12.23	-	
High grade	17.02 ± 10.42	-	

Data are shown as the mean with standard deviation or as absolute values with % in parentheses. The comparison of categorical variables (sex, smoking status) between study groups was done by the χ^2^ test, while differences in continuous variables (age, BMI) were assessed using the Mann–Whitney U test. A *p*-value < 0.05 was considered statistically significant (marked as bold). Abbreviations: BMI, body mass index; CIS, carcinoma in situ; n, number; SD, standard deviation.

**Table 2:** Association between cytokine SNPs and BC risk.

Allele/Genotype	BC (n = 73)	Controls (n = 88)	Inheritance Model	*p*-Value	OR (95% CI)
***IL6*** **rs1800795**			Allele contrast (C vs. G)	0.32	0.80 (0.51–1.24)
G	82 (56.16%)	89 (50.57%)	Codominant (GC vs. GG)	0.58	0.82 (0.36–1.89)
C	64 (43.84%)	87 (49.43%)	Codominant (CC vs. GG)	0.40	0.64 (0.24–1.70)
GG	24 (32.88%)	24 (27.27%)	Dominant (GC + CC vs. GG)	0.47	0.75 (0.35–1.62)
GC	34 (46.57%)	41 (46.59%)	Recessive (CC vs. GG + GC)	0.44	0.72 (0.31–1.67)
CC	15 (20.55%)	23 (26.14%)	Overdominant (GC vs. GG + CC)	0.99	0.99 (0.48–2.04)
			Log-additive	0.37	0.80 (0.49–1.30)
***TNF*** **rs1800629**			Allele contrast (A vs. G)	0.81	0.93 (0.50–1.72)
G	125 (85.62%)	149 (84.66%)	Codominant (GA vs. GG)	0.70	0.85 (0.36–2.02)
A	21 (14.38%)	27 (15.34%)	Codominant (AA vs. GG)	0.11	5.80 (0.53–63.39)
GG	56 (76.71%)	62 (70.45%)	Dominant (GA + AA vs. GG)	0.86	1.08 (0.48–2.42)
GA	13 (17.81%)	25 (28.41%)	Recessive (AA vs. GG + GA)	0.10	6.01 (0.55–65.35)
AA	4 (5.48%)	1 (1.14%)	Overdominant (GA vs. GG + AA)	0.61	0.80 (0.34–1.89)
			Log-additive	0.48	1.27 (0.65–2.50)
***CCL2*** **rs1024611**			Allele contrast (G vs. A)	0.19	1.42 (0.84–2.41)
A	109 (74.66%)	142 (80.68%)	Codominant (AG vs. AA)	0.48	0.78 (0.35–1.74)
G	37 (25.34%)	34 (19.32%)	Codominant (GG vs. AA)	**0.029**	5.40 (0.96–30.42)
AA	44 (60.27%)	56 (63.64%)	Dominant (AG + GG vs. AA)	0.82	1.09 (0.52–2.28)
AG	21 (28.77%)	30 (34.09%)	Recessive (GG vs. AA + AG)	**0.026**	5.82 (1.05–32.38)
GG	8 (10.96%)	2 (2.27%)	Overdominant (AG vs. AA + GG)	0.34	0.68 (0.31–1.51)
			Log-additive	0.29	1.36 (0.76–2.44)
***VEGFA*** **rs699947**			Allele contrast (A vs. C)	0.78	1.07 (0.69–1.66)
C	79 (54.11%)	98 (55.68%)	Codominant (CA vs. CC)	0.35	0.66 (0.29–1.52)
A	67 (45.89%)	78 (44.32%)	Codominant (AA vs. CC)	0.30	1.63 (0.53–5.03)
CC	21 (28.77%)	21 (23.86%)	Dominant (CA + AA vs. CC)	0.60	0.81 (0.36–1.80)
CA	37 (50.68%)	56 (63.64%)	Recessive (AA vs. CC + CA)	0.12	2.16 (0.81–5.73)
AA	15 (20.55%)	11 (15.50%)	Overdominant (CA vs. CC + AA)	0.10	0.55 (0.27–1.13)
			Log-additive	0.59	1.16 (0.67–2.01)

Allele and genotype counts are presented as absolute values with % in parentheses. Allele distribution between the study groups was compared by a χ^2^ test. Association between genotypes and BC risk was examined by a logistic regression analysis with adjustment for sex, age, smoking and BMI. A *p*-value < 0.05 was considered statistically significant (marked as bold). Abbreviations: BC, bladder cancer; CI, confidence interval; n, number; OR, odds ratio.

**Table 3:** Association of cytokine gene polymorphisms with tumor grade in BC patients.

Allele/Genotype	High Grade (n = 42)	Low Grade (n = 31)	Inheritance Model	*p*-Value	OR (95% CI)
***IL6*** **rs1800795**			Allele contrast (C vs. G)	**0.049**	0.51 (0.26–1.00)
G	53 (63.10%)	29 (46.77%)	Codominant (GC vs. GG)	0.47	0.70 (0.22–2.26)
C	31 (36.90%)	33 (53.23%)	Codominant (CC vs. GG)	0.053	0.25 (0.06–1.02)
GG	16 (38.10%)	8 (25.81%)	Dominant (GC + CC vs. GG)	0.19	0.49 (0.16–1.45)
GC	21 (50.00%)	13 (41.93%)	Recessive (CC vs. GG + GC)	**0.049**	0.30 (0.09–1.05)
CC	5 (11.90%)	10 (32.26%)	Overdominant (GC vs. GG + CC)	0.71	1.21 (0.45–3.29)
			Log-additive	0.054	0.51 (0.25–1.03)
***TNF*** **rs1800629**			Allele contrast (A vs. G)	0.051	0.40 (0.15–1.03)
G	76 (90.48%)	49 (79.03%)	Codominant (GA vs. GG)	**0.025**	0.23 (0.06–0.87)
A	8 (9.52%)	13 (20.97%)	Codominant (AA vs. GG)	0.78	0.74 (0.09–6.29)
GG	36 (85.71%)	20 (64.52%)	Dominant (GA + AA vs. GG)	**0.041**	0.30 (0.09–0.98)
GA	4 (9.52%)	9 (29.03%)	Recessive (AA vs. GG + GA)	0.95	0.94 (0.11–7.72)
AA	2 (4.76%)	2 (6.45%)	Overdominant (GA vs. GG + AA)	**0.025**	0.23 (0.06–0.88)
			Log-additive	0.12	0.50 (0.20–1.23)
***CCL2*** **rs1024611**			Allele contrast (G vs. A)	0.21	0.62 (0.29–1.31)
A	66 (78.57%)	43 (69.35%)	Codominant (AG vs. AA)	0.61	0.76 (0.24–2.37)
G	18 (21.43%)	19 (30.65%)	Codominant (GG vs. AA)	0.30	0.35 (0.07–1.81)
AA	27 (64.29%)	17 (54.84%)	Dominant (AG + GG vs. AA)	0.33	0.61 (0.22–1.67)
AG	12 (28.57%)	9 (29.03%)	Recessive (GG vs. AA + AG)	0.23	0.38 (0.08–1.89)
GG	3 (7.14%)	5 (16.13%)	Overdominant (AG vs. AA + GG)	0.82	0.88 (0.29–2.67)
			Log-additive	0.22	0.64 (0.31–1.31)
***VEGFA*** **rs699947**			Allele contrast (A vs. C)	0.41	1.32 (0.68–2.56)
C	43 (51.19%)	36 (58.06%)	Codominant (CA vs. CC)	0.65	1.30 (0.43–3.94)
A	41 (48.81%)	26 (41.94%)	Codominant (AA vs. CC)	0.30	2.16 (0.50–9.35)
CC	11 (26.19%)	10 (32.26%)	Dominant (CA + AA vs. CC)	0.46	1.48 (0.52–4.26)
CA	21 (50.00%)	16 (51.61%)	Recessive (AA vs. CC + CA)	0.34	1.83 (0.51–6.58)
AA	10 (23.81%)	5 (16.13%)	Overdominant (CA vs. CC + AA)	0.95	0.97 (0.37–2.53)
			Log-additive	0.31	1.45 (0.71–2.95)

Allele and genotype counts are presented as absolute values with % in parentheses. Allele distribution between the study groups was compared by a χ^2^ test. Association between genotypes and BC grade was examined by a logistic regression analysis with adjustment for sex, age, smoking and BMI. A *p*-value < 0.05 was considered statistically significant (marked as bold). Abbreviations: CI, confidence interval; n, number; OR, odds ratio.

**Table 4:** Association of cytokine gene polymorphisms with tumor stage in BC patients.

Allele/Genotype	pT2 + pT3 (n = 18)	pTa + pT1 (n = 55)	Inheritance Model	*p*-Value	OR (95% CI)
***IL6*** **rs1800795**			Allele contrast (C vs. G)	0.064	0.47 (0.21–1.06)
G	25 (69.44%)	57 (51.82%)	Codominant (GC vs. GG)	0.062	0.29 (0.08–1.07)
C	11 (30.56%)	53 (48.18%)	Codominant (CC vs. GG)	0.080	0.24 (0.04–1.45)
GG	9 (50.00%)	15 (27.27%)	Dominant (GC + CC vs. GG)	**0.034**	0.28 (0.08–0.93)
GC	7 (38.89%)	27 (49.09%)	Recessive (CC vs. GG + GC)	0.34	0.46 (0.09–2.46)
CC	2 (11.11%)	13 (23.64%)	Overdominant (GC vs. GG + CC)	0.19	0.46 (0.14–1.50)
			Log-additive	**0.047**	0.42 (0.17–1.03)
***TNF*** **rs1800629**			Allele contrast (A vs. G)	0.23	0.46 (0.13–1.68)
G	33 (91.67%)	92 (83.64%)	Codominant (GA vs. GG)	**0.045**	0.17 (0.02–1.46)
A	3 (8.33%)	18 (16.36%)	Codominant (AA vs. GG)	0.75	1.54 (0.13–18.97)
GG	16 (88.89%)	40 (72.73%)	Dominant (GA + AA vs. GG)	0.13	0.31 (0.06–1.61)
GA	1 (5.56%)	12 (21.82%)	Recessive (AA vs. GG + GA)	0.62	1.94 (0.16–23.40)
AA	1 (5.56%)	3 (5.45%)	Overdominant (GA vs. GG + AA)	**0.048**	0.16 (0.02–1.43)
			Log-additive	0.31	0.54 (0.15–1.94)
***CCL2*** **rs1024611**			Allele contrast (G vs. A)	**0.007**	0.20 (0.06–0.71)
A	33 (91.67%)	76 (69.09%)	Codominant (AG vs. AA)	0.17	0.39 (0.09–1.63)
G	3 (8.33%)	34 (30.91%)	Codominant (GG vs. AA)	**0.014**	0.00 (0.00–NA)
AA	15 (83.33%)	29 (52.73%)	Dominant (AG + GG vs. AA)	**0.032**	0.25 (0.06–0.99)
AG	3 (16.67%)	18 (32.73%)	Recessive (GG vs. AA + AG)	**0.023**	0.00 (0.00–NA)
GG	0 (0.00%)	8 (14.54%)	Overdominant (AG vs. AA + GG)	0.30	0.48 (0.11–2.02)
			Log-additive	**0.013**	0.27 (0.08–0.92)
***VEGFA*** **rs699947**			Allele contrast (A vs. C)	0.57	1.25 (0.59–2.65)
C	18 (50.00%)	61 (55.45%)	Codominant (CA vs. CC)	0.47	1.62 (0.41–6.36)
A	18 (50.00%)	49 (44.55%)	Codominant (AA vs. CC)	0.39	1.88 (0.35–10.02)
CC	4 (22.22%)	17 (30.91%)	Dominant (CA + AA vs. CC)	0.42	1.69 (0.46–6.24)
CA	10 (55.56%)	27 (49.09%)	Recessive (AA vs. CC + CA)	0.66	1.36 (0.34–5.37)
AA	4 (22.22%)	11 (20.00%)	Overdominant (CA vs. CC + AA)	0.71	1.23 (0.40–3.77)
			Log-additive	0.44	1.38 (0.61–3.14)

Allele and genotype counts are presented as absolute values with % in parentheses. Allele distribution between the study groups was compared by a χ^2^ test. Association between genotypes and BC stage was examined by a logistic regression analysis with adjustment for sex, age, smoking status and BMI. A *p*-value < 0.05 was considered statistically significant (marked as bold). Abbreviations: CI, confidence interval; n, number; OR, odds ratio.

**Table 5:** Association of *TNF* rs1800629 polymorphism with HRV parameters in BC patients.

Parameter	*TNF* rs1800629			Best Model	*p*-Value
	GG	GA	AA		
**SDNN (ms)**	18.49 ± 10.39	23.09 ± 10.54	38.12 ± 31.98	Log-additive	**0.0066**
**RMSSD ms)**	18.29 ± 11.79	22.54 ± 14.35	41.23 ± 43.59	Recessive	**0.011**
**DCmod (ms)**	20.57 ± 14.16	24.87 ± 15.16	46.18 ± 48.72	Recessive	**0.013**
**ACmod (ms)**	−20.18 ± 12.90	−26.53 ± 16.90	−37.80 ± 32.19	Log-additive	**0.021**
**Total power (ms^2^)**	414.10 ± 565.52	628.09 ± 559.65	1974.75 ± 2592.71	Recessive	**0.0008**
**Mean HR (beats/min)**	69.06 ± 10.05	64.38 ± 7.14	60.98 ± 8.43	Dominant	**0.040**
**PNS index**	−0.77 ± 0.80	−0.40 ± 0.87	0.37 ± 1.91	Log-additive	**0.016**
**SNS index**	2.08 ± 1.65	1.11 ± 1.26	0.54 ± 1.89	Dominant	**0.021**
**Stress index**	21.53 ± 7.51	17.27 ± 6.66	15.07 ± 9.75	Dominant	**0.035**

Data are shown as the mean with standard deviation. Association between SNP and HRV parameters was analyzed using the linear regression analysis with adjustment for sex, age, smoking and BMI. A *p*-value < 0.05 was considered statistically significant (marked as bold). Abbreviations and explanations: ACmod, modified acceleration capacity of the heart rate; BC, bladder cancer; DCmod, modified deceleration capacity of the heart rate; HRV, heart rate variability; mean HR, mean heart rate; ms, millisecond; PNS index, parasympathetic nervous system index; RMSSD, root mean square of successive differences between normal heartbeats; SDNN, standard deviation of normal-to-normal interbeat intervals; SNS index, sympathetic nervous system index; stress index, a measure of HRV reflecting cardiovascular system stress; total power, the sum of the energy in the low-frequency, high-frequency, and very-low-frequency ranges.

## Data Availability

The data presented in this study are available on request from the corresponding author due to privacy reasons.

## Supplementary Materials

The following supporting information can be downloaded at: https://www.mdpi.com/article/10.3390/ijms27083361/s1.

## Abbreviations

The following abbreviations are used in this manuscript:

ACmod	Modified acceleration capacity of the heart rate
Akt	Protein kinase B
ANS	Autonomic nervous system
BC	Bladder cancer
BMI	Body mass index
CCL2	C-C motif chemokine ligand 2
CCR2	C-C motif chemokine receptor 2
CI	Confidence interval
CIS	Carcinoma in situ
CTLA-4	Cytotoxic T-lymphocyte–associated protein 4
DCmod	Modified deceleration capacity of the heart rate
DFS	Disease-free survival
ELISA	Enzyme-linked immunosorbent assay
EMT	Epithelial–mesenchymal transition
HRV	Heart rate variability
HWE	Hardy–Weinberg equilibrium
IL-6	Interleukin-6
LAG-3	Lymphocyte activation gene-3
LMR	Lymphocyte-to-monocyte ratio
MAPK	Mitogen-activated protein kinase
MCP-1	Monocyte chemoattractant protein-1
MDSC	Myeloid-derived suppressor cells
Mean HR	Mean heart rate
MIBC	Muscle-invasive bladder cancer
MMP	Matrix metaloproteinase
NF κB	Nuclear factor kappa-light-chain-enhancer of activated B cells
NLR	Neutrophil-to-lymphocyte ratio
NMIBC	Non-muscle-invasive bladder cancer
OR	Odds ratio
PCR-RFLP	Polymerase chain reaction–restriction fragment length polymorphism
PD-1	Programmed cell death protein 1
PI3K	Phosphoinositide 3-kinase
PMR	Platelet-to-monocyte ratio
PNS	Parasympathetic nervous system
RMSSD	Root mean square of successive differences between normal heartbeats
SD	Standard deviation
SDNN	Standard deviation of normal-to-normal interbeat intervals
SNP	Single nucleotide polymorphism
SNS	Sympathetic nervous system
STAT3	Signal transducer and activator of transcription 3
TIM-3	T-cell immunoglobulin and mucin-domain containing-3
TME	Tumor microenvironment
TNF	Tumor necrosis factor
VEGF-A	Vascular endothelial growth factor A
