# Supplementary material for: Association of IL6 rs1800795, TNF rs1800629, CCL2 rs1024611 and VEGFA rs699947 Polymorphisms with Bladder Cancer Risk, Tumor Aggressiveness, and HRV Parameters of Autonomic Nervous System Regulation
Source: Int J Mol Sci. 2026 Apr 9;27(8):3361. doi: 10.3390/ijms27083361 (PMC13116236; doi:10.3390/ijms27083361)
Supplement: Supplementary file 1 [file ijms-27-03361-s001.zip › Supplementary Table S1.pdf]

Supplementary Table S1. Association between SNPs and cytokine levels in BC patients.

Genotypes	Cytokine levels	Inheritance Model	Crude Analysis		Linear Regression Analysis*	
			p-Value	Δ (95% CI)	*p*-Value	Δ (95% CI)
***IL6* rs1800795**	**IL-6 (pg/ml)**					
		Codominant (GC vs. GG)	0.69	2.21 (-2.82 – 7.24)	0.82	1.74 (-3.59 – 7.07)
		Codominant (CC vs. GG)		0.95 (-5.26 – 7.16)		1.03 (-5.40 – 7.46)
GG	13.27±5.02	Dominant (GC + CC vs. GG)	0.45	1.82 (-2.85 – 6.50)	0.55	1.50 (-3.37 – 6.38)
GC	15.48±12.78	Recessive (CC vs. GG + GC)	0.90	-0.34 (-5.80 – 5.11)	0.98	0.08 (-5.63 – 5.79)
CC	14.22±6.12	Overdominant (GC vs. GG + CC)	0.41	1.84 (-2.56 – 6.24)	0.58	1.35 (-3.37 – 6.07)
		Log-additive	0.67	0.67 (-2.39 – 3.72)	0.69	0.65 (-2.50 – 3.80)
***TNF* rs1800629**	**TNF (pg/ml)**					
		Codominant (GA vs. GG)	0.64	-8.52 (-38.49 – 21.45)	0.49	-6.29 (-36.74 – 24.16)
		Codominant (AA vs. GG)		16.82 (-29.89 – 63.53)		26.50 (-21.78 – 74.78)
GG	50.01±48.56	Dominant (GA + AA vs. GG)	0.90	-1.76 (-28.25 – 24.72)	0.87	2.19 (-24.97 – 29.36)
GA	41.48±18.42	Recessive (AA vs. GG + GA)	0.44	18.38 (-27.75 – 64.51)	0.26	27.69 (-19.91 – 75.28)
AA	66.82±62.02	Overdominant (GA vs. GG + AA)	0.52	-9.79 (-39.43 – 19.85)	0.59	-8.28 (-38.55 – 22.00)
		Log-additive	0.82	2.28 (-17.14 – 21.69)	0.56	6.00 (-13.99 – 25.99)
***MCP1* rs1024611**	**MCP-1 (pg/ml)**					
		Codominant (AG vs. AA)	0.15	69.46 (-5.58 – 144.50)	0.32	55.40 (-25.33 – 136.13)
		Codominant (GG vs. AA)		-17.81 (-117.67 – 82.05)		-20.82 (-126.58 – 84.94)
AA	350.46±118.79	Dominant (AG + GG vs. AA)	0.23	41.54 (-25.55 – 108.63)	0.41	30.05 (-40.86 – 100.97)
AG	419.92±169.13	Recessive (GG vs. AA + AG)	0.43	-39.67 (-138.53 – 59.19)	0.49	-36.81 (-140.71 – 67.09)
GG	332.65±77.24	Overdominant (AG vs. AA + GG)	0.054	72.63 (0.25 – 145.00)	0.15	58.90 (-19.24 – 137.04)
		Log-additive	0.65	10.92 (-35.78 – 57.62)	0.81	6.02 (-42.81 – 54.85)
***VEGFA* rs699947**	**VEGF-A (pg/ml)**					
		Codominant (CA vs. CC)	0.60	-4.83 (-32.60 – 22.95)	0.56	-4.93 (-33.32 – 23.46)
		Codominant (AA vs. CC)		-17.08 (-51.07 – 16.92)		-18.97 (-54.58 – 16.63)
CC	65.71±52.18	Dominant (CA + AA vs. CC)	0.54	-8.36 (-34.67 – 17.95)	0.53	-8.72 (-35.74 – 18.31)
CA	60.88±57.80	Recessive (AA vs. CC + CA)	0.34	-13.89 (-42.31 – 14.54)	0.31	-15.70 (-45.68 – 14.29)
AA	48.63±12.36	Overdominant (CA vs. CC + AA)	0.82	2.71 (-20.67 – 26.08)	0.80	3.09 (-21.01 – 27.18)
		Log-additive	0.34	-8.31 (-25.13 – 8.51)	0.31	-9.11 (-26.67 – 8.46)

Data are shown as the mean with standard deviation. Association between SNP and cytokine levels was analyzed using the linear regression analysis. **P*, Δ and 95%CI values were adjusted for sex, age, smoking status and BMI. A *p*-value <0.05 was considered statistically significant (marked as bold). Abbreviations: Δ, difference; BC, bladder cancer; CI, confidence interval
