# Supplementary material for: Association of IL6 rs1800795, TNF rs1800629, CCL2 rs1024611 and VEGFA rs699947 Polymorphisms with Bladder Cancer Risk, Tumor Aggressiveness, and HRV Parameters of Autonomic Nervous System Regulation
Source: Int J Mol Sci. 2026 Apr 9;27(8):3361. doi: 10.3390/ijms27083361 (PMC13116236; doi:10.3390/ijms27083361)
Supplement: Supplementary file 1 [file ijms-27-03361-s001.zip › Supplementary Table S3.pdf]

Supplementary Table S3. Association of *IL6* rs1800795 polymorphism with HRV parameters in BC patients.

Parameter	*IL6* rs1800795			Best model	*p*-Value
	GG	GC	CC		
**SDNN (ms)**	24.62±17.87	17.97±8.89	19.11±9.61	Dominant	0.084
**RMSSD (ms)**	24.10±21.33	18.29±12.17	18.80±11.90	Dominant	0.20
**DC mod (ms)**	26.69±24.22	20.46±14.11	21.55±14.13	Dominant	0.24
**AC mod (ms)**	-24.84±17.67	-21.17±14.72	-20.68±13.52	Dominant	0.43
**Total power (ms^2)**	825.04±1325.25	380.87±388.35	433.56±412.71	Dominant	0.064
**Mean HR (beats/min)**	67.36±9.74	69.36±10.56	64.90±7.23	Recessive	0.18
**PNS index**	-0.52±1.08	-0.78±0.91	-0.53±0.63	Overdominant	0.20
**SNS index**	1.48±1.50	2.13±1.86	1.67±1.30	Overdominant	0.16
**Stress index**	18.38±7.12	21.74±7.90	20.69±7.78	Dominant	0.20

Data are shown as the mean with standard deviation. Association between SNP and HRV parameters was analyzed using the linear regression analysis with adjustment for sex, age, smoking status and BMI. A *p*-value <0.05 was considered statistically significant (marked as bold). Abbreviations and explanations: AC mod, modified acceleration capacity of the heart rate; BC, bladder cancer; DC mod, modified deceleration capacity of the heart rate; HRV, heart rate variability; mean HR, mean heart rate; ms, milisecond; PNS index, parasympathetic nervous system index; RMSSD, root mean square of successive differences between normal heartbeats; SDNN, standard deviation of normal-to-normal interbeat intervals; SNS index, sympathetic nervous system index; stress index, a measure of HRV reflecting cardiovascular system stress; total power, the sum of the energy in the low-frequency, high-frequency, and very-low-frequency ranges
